# Discriminating Between Legitimate and Predatory Open Access Journals: Report from the International Federation for Emergency Medicine Research Committee

**DOI:** 10.5811/westjem.2016.7.30328

**Published:** 2016-08-08

**Authors:** Bhakti Hansoti, Mark I. Langdorf, Linda S. Murphy

**Affiliations:** * Johns Hopkins University, Department of Emergency Medicine, Baltimore, Maryland; † University of California, Irvine, Department of Emergency Medicine, Irvine, California; ‡ University of California, Irvine Libraries, Reference Department, Irvine, California

## Abstract

**Introduction:**

Open access (OA) medical publishing is growing rapidly. While subscription-based publishing does not charge the author, OA does. This opens the door for “predatory” publishers who take authors’ money but provide no substantial peer review or indexing to truly disseminate research findings. Discriminating between predatory and legitimate OA publishers is difficult.

**Methods:**

We searched a number of library indexing databases that were available to us through the University of California, Irvine Libraries for journals in the field of emergency medicine (EM). Using criteria from Jeffrey Beall, University of Colorado librarian and an expert on predatory publishing, and the Research Committee of the International Federation for EM, we categorized EM journals as legitimate or likely predatory.

**Results:**

We identified 150 journal titles related to EM from all sources, 55 of which met our criteria for OA (37%, the rest subscription based). Of these 55, 25 (45%) were likely to be predatory. We present lists of clearly legitimate OA journals, and, conversely, likely predatory ones. We present criteria a researcher can use to discriminate between the two. We present the indexing profiles of legitimate EM OA journals, to inform the researcher about degree of dissemination of research findings by journal.

**Conclusion:**

OA journals are proliferating rapidly. About half in EM are legitimate. The rest take substantial money from unsuspecting, usually junior, researchers and provide no value for true dissemination of findings. Researchers should be educated and aware of scam journals.

## INTRODUCTION

Dissemination of findings is the very core of every research endeavor. Publication through peer-reviewed literature educates the research community. It is also used to quantify the impact of individuals during career progression. In fact, publishing in high-quality peer-reviewed journals remains the prime metric of success for academicians, especially early career researchers focused on promotion and tenure.

Publication of research without proper scientific review is a detriment to society,^1^ can lead to unsafe/non-beneficial clinical practice, and in some cases may reward the conduct of unethical/unscientific conduct such as plagiarism, falsified data, and image manipulation.^2^–^3^ Predatory journals are motivated by financial gain, and are corrupting the communication of science.^4^ Furthermore, their main victims are primarily institutions and researchers in low- and middle-income countries (LMICs).

Predatory publishing is often confused with open access (OA), though they are distinctly different. Legitimate OA publishing benefits scientific communication, especially in LMICs, where researchers lack institutional support for access to literature.^5^ Conversely, predatory publishing upholds few if any of the best practices, yet demands payment for publishing, even from those most unable to pay.

One might argue that more OA data and research dissemination serves the public good. Furthermore, OA journals, even predatory ones, may provide an outlet for publication of papers not deemed especially timely or interesting, or showing significant positive effects. In a *British Medical Journal* (*BM*J) editorial, Jocalyn Clark and Richard Smith argue for firm action to address predatory publishers and educate researchers of the pitfalls of predatory publishing.^5^

In 2013, John Bohannon conducted a “sting” operation, to expose the lack of peer review in predatory OA journals, and published his story in the journal *Science*.^6^ He created a fictitious paper from made-up authors from non-existent African universities that purported to identify a new chemical that inhibited cancer cell growth. The paper was purposely fundamentally flawed such that any level of peer review would result in rejection from a legitimate journal. He sent the paper to 304 OA journals drawn from both the “predatory” scientific journal list of Beall (see below), and the Directory of Open Access Journals (presumably legitimate). Ultimately 157 (52%) accepted, 98 (32%) rejected it and the rest did not respond. Average time to acceptance was 40 days, and to rejection 24. He reported that 60% of those that were accepted or rejected showed no signs of peer review for content.

There is little in the peer-reviewed literature on the topic of OA publishing and submission processes.^7^ Determining whether a journal is truly predatory is difficult. One might mislabel small or nascent OA publishers lacking societal support or financial infrastructure as predatory because of their fees.

Jeffrey Beall, a librarian at the University of Colorado in Denver, has long enlightened the scientific community about predatory publishing.^8^ This paper brings disparate sources together to provide a cogent tutorial for researchers. We present examples and strategies to discriminate between legitimate and predatory OA journals. In addition, we provide guidance from the International Federation for Emergency Medicine (IFEM) Research Committee report of 2015 to educate EM researchers to avoid predatory publishers and journals.

## METHODS

A researcher from the IFEM Research Committee (BH), a journal editor (ML) and a university medical librarian (LM) collaborated to summarize the current state of potential indexing for OA journals, as well as collecting web resources to discriminate between legitimate and predatory OA journals. We defined as OA a journal that did all of the following:

1. The authors retained their own copyright to the material published.
2. The entire content of the journal was available free of charge to consumers wishing to read the articles.
3. The journal used a form of Creative Commons License.

We conducted searches between January 1 and June 10, 2016 from these directories, indexes, databases, and publishers including the US National Library of Medicine (NLM) Catalog: journals referenced in the NCBI (National Center for Biotechnology Information) Database, PubMed Central®, Google, Scimago Journal and Country Rank (SJR), Directory of Open Access Journals (DOAJ), Thomson Reuter’s Web of Science, and EBSCOhost title lists. We are confident that we identified all the world’s OA journal titles in the EM subject discipline. We used search terms: (emergencies OR emergency OR ER) AND (open access OR OA OR PMC) to pare these down to only OA EM-related journals.

Google works with publishers to index articles, theses, preprints, book chapters, abstracts, and technical reports from all research disciplines and make them searchable on Google Scholar. However, while these services readily retrieve individual papers, it is challenging to retrieve EM journal titles. We performed a comprehensive Google search, and then pared results to identify a list of OA EM journals. We then removed duplicates, included only journals that are 100% OA, and publish in English. We excluded any partial OA (a stated embargo period during which readers would need to pay for access) or inactive titles.

We then scrutinized the journals, searching their websites and applying Beall’s criteria to identify and clarify which were likely to be “predatory” journals.^8^ We drew on collective experience in medical publishing and journal editing to identify features and characteristics that suggested that journals were primarily interested in profit over science, and lacked features inherent in legitimate journals in the field. A journal was categorized as likely predatory when it was not included in any recognized medical library indexing services (beyond Google), and did not have features on its website of legitimate OA journals. We present the journal titles and their website links as aides to authors who wish to further scrutinize the characteristics of a journal before submission.

As this paper did not involve human subjects, approval was not obtained.

## RESULTS

We present our comprehensive findings of OA journals that relate to EM, and are likely to be either legitimate or predatory. We first divide our results by each index we searched to identify OA EM journal titles. We then present journal titles (Table 1) from Beall’s list of predatory OA journals that relate to EM. Next, we present indexing services where legitimate open access journals may be found, and the number of journals indexed with the word “emergency”, “emergencies” or “ER” in the title, and notation of how many are OA (Table 2).

Next, we present a list of OA journals with the word “emergency” in the title that are archived in PubMed Central with full paper deposition and no or one-month embargo period (Table 3). These are clearly legitimate by virtue of their inclusion in PubMed.

Then, we present a list of legitimate OA journals in EM, along with the well-recognized indexing services in which they are contained (Table 4). By virtue of their inclusion in indexing services, they are legitimate OA journals. Finally, we present a list of likely predatory OA journals in EM (Table 5) that are not included in any reputable index. Therefore, Tables 1 and 5 are likely predatory OA journals, while Tables 3 and 4 list legitimate EM OA titles.

The three authors agreed with the designation of OA journal in all cases. After scrutiny of the websites, this became very clear by the description, copyright agreement and Creative Commons licenses. The authors also were entirely in agreement regarding the titles listed in Tables 3 (PubMed Central indexed) and 4 that these were legitimate OA journals. All others were highly suspected of being predatory.

### NLM Catalog Search for OA Journals in EM

The United States (US) NLM Catalog includes journals in all NLM collections and databases (e.g., PubMed, PubMed Central, MEDLINE, Nucleotide, Protein, etc). We found 127 titles in PubMed, PubMed Central, and MEDLINE, but only 17 are fully OA and contain the word “emergency,” “emergencies,” or “ER” in their title. See Table 2.

### PubMed Central Journal list in EM

PubMed Central is a US NLM repository and archive of OA full-text (not just abstract) scholarly articles. Our search identified 17 journals that are in “full participation” of OA. One title, “*California Journal of Emergency Medicine*” changed its name to “*Western Journal of Emergency Medicine*” and has remained fully OA. Two titles dropped their names and merged with a new name (*Emergency Medicine Journal* or *EMJ*). Since then, only a few selected articles are open access (Table 2), leaving 14 of the 17 journals that are truly OA. These journals must meet rigid scientific quality standards (http://www.ncbi.nlm.nih.gov/pmc/pub/addjournal/) to be included in PubMed Central. Therefore, they are all clearly legitimate and not predatory journals.

### SJR (Scimago Journal & Country Rank) Journal Subject Category Search

The SJR is a mathematical analysis designed to measure the relative impact of journal articles by their citation frequency and importance, and then to rank the journals themselves within scientific fields. In the SJR website (http://www.scimagojr.com/journalrank.php?area=2700&category=2711), we selected “Medicine” as the subject area and “Emergency Medicine” as the subject categories in all regions and countries for 2015 journals. The results returned 72 journals. We then selected the “Display only Open Access Journals” box, yielding 17 titles, but only 12 were fully OA journals that met our inclusion criteria (Table 2).

### Directory of Open Access Journal (DOAJ) Search

In the DOAJ homepage, we deselected “articles” and searched only for journal titles with a truncation search for emergenc^*^. The results returned 46 OA journals that contained the word “emergency” or “emergencies” in the title, but only 19 met our inclusion criteria as fully OA (Table 2).

### Thomson Reuters Master Journal List Search

Thomson Reuters is a major multinational mass media and information firm headquartered in Toronto and New York. Its business is to select, index and sell access to scholarly works to university libraries. The company includes select journals in its indexes upon application, but rejects many titles that do not meet its standards. The Thomson Reuters Master Journal List includes all journals indexed in their propriety product called “Web of Science.” This includes the Emerging Sources Citation Index, created in 2015 (http://wokinfo.com/products_tools/multidisciplinary/esci/). With our truncated search for title word, “emergenc^*^,” we located 33 titles in EM, with 11 of them fully OA (Table 2).

### EBSCOhost Title Lists Search

EBSCO, headquartered in Ipswich, Massachusetts, is a private company that indexes and sells access to scholarly works to academic and medical libraries (and many other products) through 375 databases. EBSCOhost houses titles by subject category. We reviewed and included only two relevant EBSCO databases, CINAHL (Cumulative Index to Nursing and Allied Health Literature) and Academic Search Complete that would most likely index EM titles. Our reviews identified 63 journals that contain the word “emergency” or “emergencies” in the title, but only 10 qualified as completely OA (Table 2).

### Google Search for OA Journals in EM

In Google, we searched for “Open Access Emergency Medicine Journal” (without the quotation marks) and examined the first 15 pages of results. These contained 150 sites that were linked to library journal directories, publishers and independent journal websites. After thorough review, we identified 57 (38%) initially determined to be OA journals. Two were excluded (leaving 55, or 37% OA) as known EM magazines (*EM World* and *EM Resident*) that would not be expected to achieve indexing or undergo formal peer review. These were then compared with titles from established indexing services worldwide described above (Table 2).

One of the major markers of a predatory OA journal is the absence of indexing in any recognized reference index. Twenty-five of these 55 titles (45%, Table 5) were not included in any index, and therefore are highly suspicious of being predatory. Alternatively, they could be very early in their development and therefore cannot offer wide discovery in established search services. For example, we identified two journals that, while not included in any of the indexes we searched, nevertheless are legitimate and OA according to examination of their websites (*Japanese Journal of Trauma and Emergency Medicine,* an inactive title last published was in 2012, and *Emergency Medicine and Health Care).*

### Beall’s Predatory OA list

We found that the most recent list of predatory OA journals from Beall updated January 7, 2016, contains 923 publishers from all scientific fields, each of which publishes multiple (3–30+) journals (33% increase over 2015) and 882 stand-alone journals (74% increase over 2015).^8^ Fortunately, none of the 923 publishers contain the word “emergency” or “medicine” in their titles, and none of the 882 stand-alone journals contain the stem, “emerg” (except for the word “emerging” in many journal titles).

We did identify 16 “medical” journals among the 882 stand-alones. Table 1 provides a short list of journals that may appear related enough for a novice researcher to be fooled into submitting an EM article (taken from the large list by one of the authors, ML). For perspective, the largest of the biomedical research legitimate journal databases (HINARI Program from the World Health Organization) lists 14,964 journal titles worldwide, so these “predatory” journals amount to 12–50% of the legitimate titles (calculating 1–7 journals per predatory publisher, which appears to be the case from Beall’s list).

### Indexing Services

The major biomedical indexing services identified by the authors are listed in Table 2, with the number of journals (OA/total including subscription) with the word “emergency,” “emergencies” or “emerg^*^” in their titles.

## DISCUSSION

Given this disturbing trend in OA publishing, the IFEM Research Committee sought to provide guidelines to identify potential predatory journals, and summarize Beall’s criteria for determining predatory OA publishers. We present the report here:

### Overall Approach to Choosing the Journal

1. Choose to submit your research to journals that you would normally find interesting and relevant. Although these may be among the most discriminating, if successful, this will increase the chance that your research will be disseminated to the community you want to reach. This may result in changes to practice or policy, providing the most impact for your work.
2. Reach out to colleagues and mentors to see in which journals your body of work will best fit. Mentors have an understanding of historical trends in the scientific community in general, and EM specifically. The university librarian can be a valuable resource to find legitimate impact factors of the journals you are considering, and the distribution of these journals to academic communities. Beware, however, that impact factors (as well as peer-review processes) can be fabricated. Beall published a new list in 2015 of 38 “Misleading Metrics” companies that purport to gauge a journal’s impact factor, and provide scholarly metrics at the researcher and article level.^10^
3. Be honest about the methodological flaws of your own work. It is unlikely that good reviewers will not identify them. If the reviewers do not see the same limitations as you have, this is a red flag that you have sent your work to a predatory journal. Be concerned if you do not receive any critical feedback and your article is accepted, as this rarely happens with legitimate peer review.
4. Look up the journal title in the NLM Catalog: Journals referenced in the NCBI Databases.^11^ If the journal is found there, you can review the detail record where it provides information about the journal and whether the journal is indexed in any of the NCBI databases such as MEDLINE, PubMed, or PubMed Central. PubMed Central is a free digital repository that archives publicly accessible full-text scholarly articles that have been published within the biomedical and life sciences journal literature. Journal acceptance to PubMed Central went through a vetting process. These journals may also be indexed in MEDLINE, though this index is substantially more discriminating. However, OA journals whose content is listed in PubMed Central (full papers) automatically have their abstracts migrated to the PubMed.

However, this does not guarantee legitimacy of the journal. In the present study separate from the IFEM Research Committee guidance, we found the journal, *Emergency Medicine: Open Access*, in the NLM Catalog. It further shows that the journal was found in PubMed and PubMed Central. However, after thorough review, we found only one citation in PubMed (PMID: 25035816) as well as PubMed Central (PMC: 4098070) for the entire journal. This occurred when the authors of the paper from Taiwan chose to deposit the article to PubMed Central.

5. If claiming to be an OA journal, is it in the Directory of Open Access Journals (DOAJ) ? This is a sort of “whitelist” of legitimate journals that must meet specific criteria for inclusion.^12^
6. Is the journal transparent and following best practice in editorial and peer-review processes, governance, and ownership? The best way to discern this is by reviewing the journal documents and governance, likely available online at the website, or reaching out to the journal leadership. Legitimate journals should have a robust list of policies and procedures on their website, including human and animal subject policies, OA license type (something like Creative Commons Attribution License 4.0), conflict of interest and informed consent.
7. Read the articles in the journal before submitting an article. Warning signs include grammar errors, poor quality science, poorly maintained website with prominent misspellings and grammatical errors.
8. Is the name of the journal incongruent with the journal’s mission? Is the name of the journal excessively broad? Does the name of the journal make sense?
9. Are there clear policies on plagiarism, authorship, and copyright on the website?
10. Is the impact factor clearly stated? Is it too good to be true (> 2)? If not readily available, journal impact factors can be found in the ISI Thomson Reuters Journal Citation Reports (JCR),^13^ which requires institutional subscription, and the Scimago Journal & Country Rank^9^ which is free.
11. Is the journal found in PubMed Central? PubMed Central allows publishers to deposit their OA journal contents for permanent archival without cost upon application and fulfilling features of legitimate and respectable journals for two years of publishing.^11^

### The Publisher

Identify if the publisher is genuine. This requires some research evaluating the publisher’s content practices and websites. Below are some of the aspects of the journal that may provide clues to a predatory publishing journal.

1. Submit work to publishers with enlightened copyright policies. Determine whether you can post and share your work once published. One of the main advantages of OA publishing is that the author maintains their own copyright, and therefore ability to use the material in other scholarly works. SHERPA/RoMEO^14^ collects information on default copyright and self-archiving policies for both publishers and individual journals.
2. Complete an analysis of the publisher’s content, practices, and websites according to ethical standards established by membership organizations. Numerous organizations have provided comprehensive ethical standards for publishers. Three are listed below.
  - Open Access Scholarly Publishers Association (OASPA)^15^
  - Committee on Publication Ethics (COPE)^16^
  - International Association of Scientific, Technical & Medical Publishers (STM). 17

Consider asking the following questions when reviewing the publisher’s website.

1. Does the journal clearly identify an editor-in-chief and an editorial board?
2. Do the editors/editorial review board have academic affiliations and appropriate credentials for the journal scope and topic?
3. Does it provide specific and detailed instructions and guidelines for authors?
4. Are policies and practices fully stated?
5. Are author fees reasonable and are they clearly stated up front? OA fees to the author range from $300 to more than $4,000 USD. Wolters Kluwer, a legitimate publisher, openly lists fees for hundreds of their journals on their website.^18^
6. Do they offer discount or waiver for junior authors or authors from LMICs who can’t afford to pay the author fee?
7. Can you find the publication fee easily identified on the website, or is it hidden many screens back with obscure navigation?
8. Does the publisher ask the corresponding author for suggested reviewers? This is, in general, a negative feature of a journal, and implies it does not have enough legitimately qualified and dedicated reviewers to perform this important scientific service to the profession or specialty.
9. Does the publisher engage in excessive use of spam emails to submit manuscripts? Specific targeted requests for papers to an established author are legitimate, and they come one at a time from the editor. Conversely, blanket solicitations addressed to “esteemed author” and similar salutations are markers of predatory OA journals.
10. Do the publisher’s officers use email addresses that end in .gmail.com, .yahoo.com, or some other free email supplier?
11. Does the publisher have excessive advertising on the website?
12. Does the publisher have no membership or industry association? In particular, legitimate journals have membership in multiple indexes.

In truth, it can be difficult to distinguish between legitimate and non-legitimate journals. Beall has written about predatory open access journals since at least 2011. His website now has a section on “Hijacked Journals,” which lists predatory/fake journals meant to look and sound like the titles of legitimate ones.^19^ In our findings, two journals related to EM have apparently been hijacked. “*Emergencias,*” the Spanish language legitimate journal has a fake journal by the same name. A predatory journal, *OA Emergency Medicine* imitates a legitimate journal, *Open Access Emergency Medicine* from Dovepress. “Hijacked” journals are further challenging to decipher given their ability to mimic legitimate peer-review outlets.^7^, ^20^–^21^

Within Beall’s list in 2015, we identified only nine journal titles from medicine that might tempt an EM author to submit (Table 1). However, the number of predatory journals and publishers is expanding weekly, so additional titles are now just as problematic. With difficulty and the guidance of a medical librarian, we identified multiple sources and indexes that contain EM journals, both subscription and OA. Of 150 titles, we found that about a third were OA, and of these half were predatory. However, per Beall, the number of predatory publishers and individual journal titles is proliferating exponentially in all scientific fields, from 18 publishers in 2011 to 923 listed in 2016, and from 126 stand-alone journals in 2013 to 882 in 2016. Clearly, lists are insufficient to guide authors. Only an understanding of the practices and markers of legitimate and predatory OA publishers will allow the researcher to keep pace with danger.

Tables 1 and 5 present likely predatory OA journals, while Table 3 presents clearly legitimate OA journals indexed in PubMed, which requires a high standard application from publishers. Table 4 shows legitimate OA journals and allows the reader of this piece to determine how widely an EM OA journal is indexed. This, in turn, determines how easy it would be for a reader or researcher interested in this subject to find their work.

Even with the guidance contained in the IFEM Research Committee report, and the list of likely predatory journals above, it can be difficult to identify predatory OA journals. Increasing numbers of “predatory publishers” take advantage of the “pay to publish” model used by legitimate OA publishers such as BioMed Central (https://www.biomedcentral.com/) and PLoS (https://www.plos.org/ Public Library of Science). Legitimate OA publishers charge authors to cover operational costs and management of peer review. These predatory OA publishers request high fees in exchange for quick, OA publication online, without robust editorial and publishing services or widespread indexing. Peer review is cursory at best, or sham at worst. Unfortunately, junior scholars at even well-resourced universities and scientists/institutions in LMIC are most at risk for these practices. These researchers may unknowingly submit to predatory journals, only then receive an invoice after the paper is “accepted,” and not have the means to pay the fee.

Traditionally, librarians were the sophisticated gatekeepers to scientific information and advised researchers regarding mainstream journals. But with search engines such as Google Scholar©, researchers may find it difficult to distinguish between reputable and predatory publishers. All are found there, whether or not there has been any quality control. Nevertheless, consultation with a medical or science librarian, when in doubt, can clarify the status of an OA journal.

An additional negative feature of predatory publishers is that they can refuse to retract articles at author request, when their true nature is discovered. In the *BMJ* editorial and blogs, Clark stresses that, once a paper is published in one of these predatory journals, they are not cooperative with attempts to “retract,” protesting that theirs is a legitimate outlet for science.^22^ She therefore advocates that institutions create “acceptable journals lists” to distinguish the differences. The medical librarian at each institution can play a major role in creating such a list. She also advises authors to stick with journals in the specialty that are more widely known.^23^

## LIMITATIONS

We did not include words such as “trauma” and “disaster” and their variations in our searches. As a result, our study did not include possible predatory/hijacked journals that contain those words in the journal titles, e.g., *Austin Journal of Trauma and Treatment* (https://scholarlyoa.com/2016/03/03/a-true-predator-austin-publishing-group/). In addition, future research is needed to determine the true nature (legitimacy) of each non-indexed OA journal that we identified in Table 5. In addition, we may have included legitimate journals that are simply not indexed in any of the databases we searched. For example, we know that *Emergency Medicine and Health Care* (http://www.hoajonline.com/includes/homepages/search.php?searchtype=&searchtext=emergency+medicnie+and+health+care&journal%5B%5D=22&articletype=&PageSize=10&search=search) and the *Japanese Journal of Trauma and Emergency Medicine* (https://www.jstage.jst.go.jp/browse/jjtem) are legitimate, reputable journals that were found in our Google search, but were not indexed in any of the databases/directories that we selected for review. The landscape of publishing changes weekly, so there may be new legitimate and OA journals. Finally, we only searched for journals in English.

## CONCLUSION

The novice scholar in EM should be cautious when submitting work to OA journals. This article educates and enables discrimination between legitimate and predatory OA journals. Legitimate ones disclose publishing fees freely, perform meaningful peer review, and then disseminate scholarship to the world without cost to the reader. Predatory OA journals hide fees, disclose them after acceptance, perform sham peer review or none at all, accept (nearly) all submissions to generate revenue, do not cooperate with publication retractions, and are not indexed sufficiently to allow other researchers to find the work. The number of these predatory journals and publishers is expanding rapidly, and threatens the integrity of scientific research and publishing.

## Figures and Tables

**Table 1:** Journal titles on Beall’s Predatory Open Access list, 2015, containing the word, “medicine” in the title that may appear related enough to submit an emergency medicine article.

1	Global Journal of Medicine and Public Health
2	Hygeia: Journal for Drugs and Medicines
3	Internal Medicine Review (has not published any articles)
4	International Journal of Collaborative Research on Internal Medicine & Public Health (IJCRIMPH)
5	International Journal of Medicine and Biosciences
6	Journal of Advances in Internal Medicine
7	Journal of Coastal Life Medicine
8	Journal of Evidence Based Medicine and Healthcare (JEBMH)
9	Translational Medicine and Biotechnology (TMB)

**Table 2:** Indexing services where legitimate open access journals may be found, and the number of journals indexed with the word “emergency”, “emergencies” or “emerg^*^” in the title, with denominators representing total number of journal titles, including subscription journals. Numerator = OA.

Index name	Website link	Number of OA journals with “emergency” “emergencies” or “emerg^*^” in title
The NLM Catalog	http://www.ncbi.nlm.nih.gov/nlmcatalog/journals	17/127
PMC Journal List	http://www.ncbi.nlm.nih.gov/pmc/journals/	14/17
EBSCOhost Title Lists	https://www.ebscohost.com/title-lists This list includes titles from CINAHL and Academic search complete	10/63
SJR	http://www.scimagojr.com/journalrank.php This list includes Scopus and EMBASE titles.	12/72
DOAJ (Directory of Open Access Journals)	https://doaj.org/search	19/46
Thompson Reuters Web of Science Master Journal Lists	http://ip-science.thomsonreuters.com/mjl/	11/33
Google search	https://www.google.com/	55/150

*NLM*, National Library of Medicine; *PMC*, PubMed Central; *EBSCOhost*, Elton B. Stephens Company; *SJR,* Scimago Journal Rank; *DOAJ*, Director of Open Access Journals; *EMBASE*, Excerpta Medica Database

Scopus has no abbreviation.

**Table 3:** List of open access journals with the word “emergency” in the title that are indexed in PubMed Central with full paper deposition and no or one-month embargo period.

ISSN	Title	Latest	First	Free Access	Participation Level
0264-4924	Archives of Emergency Medicine — now published as Emergency Medicine Journal : EMJ	v.10(4) Dec 1993	v.1 1984	Immediate	Full
1471-227X	BMC Emergency Medicine	v.16 2016	v.1 2001	Immediate 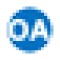	Full
2322-2522	Bulletin of Emergency & Trauma	v.4(2) Apr 2016	v.1 2013	Immediate	Full
1948-3384	The California Journal of Emergency Medicine — now published as Western Journal of Emergency Medicine	v.8(2) May 2007	v.1 2000	Immediate 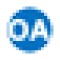	Full
2090-648X	Case Reports in Emergency Medicine	v.2015 2015	v.2011 2011	Immediate 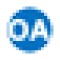	Full
2345-4563	Emergency	v.3(4) Autumn 2015	v.1 2013	Immediate 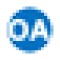	Full
2090-2840	Emergency Medicine International	v.2015 2015	v.2010 2010	Immediate 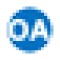	Full
1472-0205	Emergency Medicine Journal : EMJ (v.1;1984)	v.24(12) Dec 2007	v.18 2001		Now Select only (Full)
1865-1372	International Journal of Emergency Medicine	v.8 2015	v.1 2008	Immediate 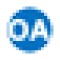	Full
1351-0622	Journal of Accident & Emergency Medicine — now published as Emergency Medicine Journal : EMJ	v.17(6) Nov 2000	v.11 1994	Immediate	Full
0974-2700	Journal of Emergencies, Trauma, and Shock	v.9(2) Apr–Jun 2016	v.1 2008	Immediate 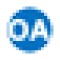	Full
1179-1500	Open Access Emergency Medicine : OAEM	v.8 2016	v.1 2009	Immediate 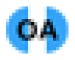	Full
1757-7241	Scandinavian Journal of Trauma, Resuscitation and Emergency Medicine	v.24 2016	v.16 2008	Immediate 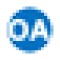	Full
2452-2473	Turkish Journal of Emergency Medicine	v.16(1) Mar 2016	v.14 2014	Immediate 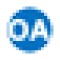	Full
1936-900X	Western Journal of Emergency Medicine (v.1;2000)	v.17(1) Jan 2016	v.8 2007	Immediate 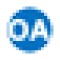	Full
1920-8642	World Journal of Emergency Medicine	v.6(4) 2015	v.1 2010	1 month 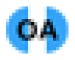	Full
1749-7922	World Journal of Emergency Surgery : WJES	v.11 2016	v.1 2006	Immediate 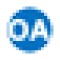	Full

*ISSN*, International Standard Serial Number; *v*, volume

**Table 4:** Open access emergency medicine journals that have achieved indexing in recognized services and are therefore legitimate rather than predatory.

	Journal Title and Weblink	NLM Catalog	PubMed Central	SJR	DOAJ	EBSCOhost	WS
1	Advances in Emergency Medicine from Hindawi http://www.hindawi.com/journals/aem/contents/				X		
2	African Journal of Emergency Medicine http://www.afjem.org/	X	X	X	X		X
3	BMC Emergency Medicine https://bmcemergmed.biomedcentral.com/	X	X	X	X	X	X
4	Bulletin of Emergency & Trauma http://www.beat-journal.com/BEATJournal/index.php/BEAT	X	X		X		
5	Case Reports in Emergency Medicine from Hindawi Publishing Corporation http://www.hindawi.com/journals/	X	X		X		
6	EAJEM: Eurasian Journal of Emergency Medicine http://www.akademikaciltip.com/eng/Anasayfa						X
7	Emergency: An Academic Emergency Medicine Journal http://journals.sbmu.ac.ir/emergency	X	X		X		
8	Emergency Care Journal http://www.pagepressjournals.org/index.php/ecj				X		X
9	Emergency Medicine International from Hindawi Publishing Corporation http://www.hindawi.com/journals/emi/	X	X		X	X	X
10	Emergency Medicine: Open Access* http://www.omicsgroup.org/journals/emergency-medicine.php	X					
11	Hong Kong Journal of Emergency Medicine http://www.hkjem.com/			X			X
13	International Journal of Emergency Medicine from Springer Open https://intjem.springeropen.com/	X	X	X	X	X	X
14	International journal of Emergency Mental Health and Human Resilience http://www.omicsonline.com/open-access/international-journal-of-emergency-mental-health-and-human-resilience.php	X		X			X
15	Iranian Journal of Emergency Medicine http://www.journals.sbmu.ac.ir/en-iranjem				X		
18	ISRN Emergency Medicine http://www.hindawi.com/journals/isrn/contents/emergency.medicine/				X	X	
19	Journal of Cardiovascular Emergencies http://www.degruyter.com/view/j/jce				X		
20	Journal of Emergencies, Trauma, and Shock http://www.onlinejets.org/	X	X	X	X	X	
21	Journal of Emergency Medicine, Trauma and Acute Care from Qscience.com http://www.qscience.com/loi/jemtac			X	X		
22	Journal of Emergency Practice and Trauma http://jept.ir/				X		
23	Journal of Trauma Management and Outcome https://traumamanagement.biomedcentral.com/			X			
24	Open Access Emergency Medicine from Dovepress https://www.dovepress.com/open-access-emergency-medicine-journal	X	X	X	X	X	X
25	Scandinavian Journal of Trauma, Resuscitation and Emergency Medicine http://sjtrem.biomedcentral.com/	X	X	X	X		X
26	Turkish Journal of Emergency Medicine http://www.trjemergmed.com/	X	X				X
27	Western Journal of Emergency Medicine: Integrating Emergency Care with Population Health http://escholarship.org/uc/uciem_westjem	X	X	X	X	X	X
28	World Journal of Emergency Medicine http://www.wjem.org/	X	X				
29	World Journal of Emergency Surgery http://wjes.biomedcentral.com/	X	X	X	X		

*NLM*, National Library of Medicine; *SJR*, Scimago Journal Rank; *DOAJ,* Director of Open Access Journals; *WS*, Thomson Reuters Web of Science

Emergency Medicine: Open Access is not indexed in PubMed. Only one citation was found in PubMed.

**Table 5:** Emergency medicine journals that have not achieved indexing in any recognized service and are therefore likely predatory.

	Journal Title and Weblink
1	Archives of Emergency Medicine and Critical care http://www.jscimedcentral.com/EmergencyMedicine/
2	Austin Emergency Medicine http://austinpublishinggroup.com/emergency-medicine/
3	Australian Journal of Emergency Management https://ajem.infoservices.com.au/items/AJEM-31-02
4	Clinical Experimental Emergency Medicine http://ceemjournal.org/about/index.php
5	Edorium Journal of Emergency Medicine http://www.edoriumjournalofemergencymedicine.com/about-us/about-us.php
6	Emergency Medicine and Health Care from HOAJ (Herbert Open Access Journal) http://www.hoajonline.com/emergmedhealthcare http://www.hoajonline.com/)
7	Emergency Medicine Open Journal from Openventio Publishers http://openventio.org/OpenJournal/EmergencyMedicine.html http://openventio.org/index.php
8	Frontiers in Public Health | Disaster and Emergency Medicine http://journal.frontiersin.org/journal/public-health/section/disaster-and-emergency-medicine
9	Gavin Journal of Emergency Medicine (journal has not published any issues) http://gavinpublishers.org/index.php/emergency-medicine
10	Henry Journal of Emergency Medicine, Trauma & Surgical Care (journal has not published any issues) http://www.henrypublishinggroup.com/index.php/emergencymedicine/about
11	HSOA Journal of Emergency Medicine, Trauma & Surgical Care http://www.heraldopenaccess.us/journals/Emergency-Medicine-Trauma-&-Surgical-Care/
12	International Journal of Critical Care and Emergency Medicine http://clinmedjournals.org/International-Journal-of-Critical-Care-and-Emergency-Medicine.php
13	Internet Journal of Emergency Medicine http://ispub.com/IJEM from Internet Scientific Publications.
14	Journal of Emergency Medicine & Critical Care from Avens Publishing Group Inviting Innovations http://www.avensonline.org/medical/emergency-medicine-and-critical-care/home-5/
15	Journal of Emergency Medicine and Intensive Care http://elynsgroup.com/journal/journal-of-emergency-medicine-and-intensive-care
16	Journal of General and Emergency Medicine http://scientonline.org/journals/general-emergency-medicine/31
17	Journal of Intensive Care and Emergency Medicine http://www.signavitae.com/
18	Mathews Journal of Emergency Medicine http://www.mathewsopenaccess.com/EMedicine.html
19	OA Emergency Medicine http://www.oapublishinglondon.com/oa-emergency-medicine
20	Open Emergency Medicine (latest content is 2013) http://benthamopen.com/toemj/home
21	Open Journal of Emergency Medicine from Scientific Research An Academic Publisher http://www.scirp.org/journal/ojem/
22	Pediatric Emergency Care and Medicine: Open Access http://pediatric-emergency-care.imedpub.com/
23	The Scientific Pages of Emergency Medicine http://thescientificpages.org/page/general-medicine/scientific-pages-of-emergency-medicine.php
24	SM Emergency Medicine and Critical Care (SMEM) http://smjournals.com/emergency-medicine/
25	Trauma and Emergency Care (TEC) from OAT (Open Access Text) http://oatext.com/Trauma-and-Emergency-Care-TEC.php
